# Evaluation of Fluid Loss and Customary Fluid Intake among a Selected Group of Young Swimmers: A Preliminary Field Study

**DOI:** 10.3390/ijerph18063205

**Published:** 2021-03-19

**Authors:** Damian Wiśniewski, Ewa Śliwicka, Jakub Malik, Krzysztof Durkalec-Michalski

**Affiliations:** 1 Department of Sports Dietetics, Poznan University of Physical Education, 61-871 Poznań, Poland; wisniewski@awf.poznan.pl; 2 Department of Physiology and Biochemistry, Poznan University of Physical Education, 61-871 Poznań, Poland; sliwicka@awf.poznan.pl; 3 Department of Physical Activity and Health Promotion Science, Poznan University of Physical Education, 61-871 Poznań, Poland; malik@awf.poznan.pl; 4 Department of Human Nutrition and Dietetics, Poznan University of Life Sciences, 61-871 Poznań, Poland

**Keywords:** hydration, dehydration, body composition, training, young athletes, aquatic sports

## Abstract

This study aimed to assess fluid loss (FL) and customary fluid intake (FI) during a training session, and the relationship between FL and total body water (TBW) content in a selected group of young swimmers. The study involved 17 (seven females, 10 males) individuals whose anthropometric and body composition analyses and FI during training units were carried out. The total average FI and total actual FL oscillated around 531 mL and −513 mL for the whole study group (469 mL and −284 mL for females, 574 mL and −674 mL for males). The dependent and independent sample *t*-tests, the Cohen’s d effect size and Pearson’s correlation coefficient were analysed. Significant differences were observed between pre-workout and post-workout body weights after training without FI in the whole group (66.5 kg vs. 66.0 kg, *p* < 0.001, d = 0.06), in females (61.2 kg vs. 60.9 kg, *p* = 0.015, d = 0.04) and males (70.3 kg vs. 69.6 kg, *p* < 0.001, d = 0.9). For the TBW content and fat-free mass (FFM) before and after training, significant differences were observed only in males (TBW: 43.8 L vs. 43.2 L, *p* = 0.002, d = 0.14; and 62.4% vs. 61.7%, *p* < 0.001, d = 0.36; FFM: 59.8 kg vs. 59.1 kg, *p* = 0.002, d = 0.12). Moreover, the relationship between the actual FL and TBW before training was observed in the whole (mL vs. %: r = −0.64, *p* = 0.006; mL vs. L: r = −0.84, *p* < 0.001) and the male group (mL vs. L: r = −0.73, *p* = 0.017). These results indicated FL in young swimmers during training and the relationship between FL and pre-training TBW content, which suggests that it is important to also pay special attention to effective hydration procedures before and during training in aquatic environments.

## 1. Introduction

Swimming has been included in the Olympic disciplines since the first modern Summer Olympics, which took place in 1896 [1]. Currently, the swimming competitions are held in swimming pools of 50 and 25 m in length. The most important swimming competitions, e.g., during the Olympic Games or the World Championships, are held in a pool (the so-called “Olympic Pool”) that is 50 (length) × 25 (width) m, a minimum of two meters deep and divided into 10 tracks. The water temperature should oscillate between 25 and 28 °C [2]. It should be noted that in an aquatic environment (such as in swimming disciplines), the human organism is subject to different environmental conditions than when it is on land. Among other things, it is affected by high hydrostatic pressure, which increases blood flow to the main body organs (brain, heart, lungs) and stimulates diuretic activity [3]. It remains unclear how the water environment (especially during exercise) influences sweating specificity. This can be crucial in sports because sweating is the most efficient mechanism for maintaining the optimal body temperature, and facilitating the removal of some metabolic products [4].

It should therefore be emphasised that proper hydration is vital because water in the human body, apart from its thermoregulatory role, also determines many other functions, such as transporting nutrients and giving structure to cells and tissues [5]. It is claimed that the human body in natural conditions could function without fluid intake (FI) for only two to four days [5]. Furthermore, it is estimated that the total body water (TBW) content in the human body oscillates on average around 59% in the case of men, and about 56% in the case of women [6], but these discrepancies are primarily related to body composition differences [7]. Moreover, in adolescents, TBW usually ranges from 49 to 63% in females and 52 to 66% in males, and these values decrease with age [8].

However, when the relationship between FI and FL (fluid loss) is unbalanced, dehydration may occur, such as a reduction in TBW due to FL, reduced FI or both [5]. It should be noted that dehydration may significantly and very quickly undermine the optimal functioning of the organism by, among others, deteriorating the mood, disrupting cognitive functions or reducing glycaemic regulation [9]. This condition may be a pathogenic factor in such diseases and conditions as hypertension, coronary artery disease or stroke [10]. Dehydration is also a serious problem in sport, impairing physical capacity and exercise performance in a wide range of disciplines [11,12,13,14,15,16]. It must be underlined that inadequate body hydration status leads to cardiorespiratory disturbances [17], a higher frequency of heart contractions at sub-maximum load [11], elevated lactate concentration during long-term efforts [12], a higher degree of perceived exercise load and lower power output during time trials [13], and lower maximum oxygen uptake (VO_2_max) [14] in exercising subjects.

Finally, the problem of dehydration risk seems to be crucial in young people. The prevalence of inadequate hydration status among 4134 participants aged 6 to 19 years even reached 54.5% [18]. The severity of this problem can be exacerbated by environmental conditions such as high temperature. Research conducted in Cyprus revealed that ninety percent of the adolescents (141 participants aged 15 to 17 years) arrive customarily dehydrated at school [19]. This issue is also observed in young athletes in different sports (including young swimmers) [20,21]. For example, Adams et al. [21] tested osmolality and specific gravity in morning urine samples, and recorded that 67% of young swimmers started their first training unit in an inadequate state of hydration. Demonstrated irregularities in hydration status may lead to negative exercise and health consequences [22], which proves the need to conduct research in this area. In our studies, we hypothesised that the FI during usual training units would be low, which would result in a significant reduction in body mass due to FL related to exercise-induced thermoregulatory activity. For these reasons, this study aimed to assess (1) fluid loss (FL) and customary fluid intake (FI) during a training session, and (2) the relationship between FL and total body water (TBW) content in a selected group of young swimmers.

## 2. Materials and Methods

### 2.1. Participants

**Figure 1:**
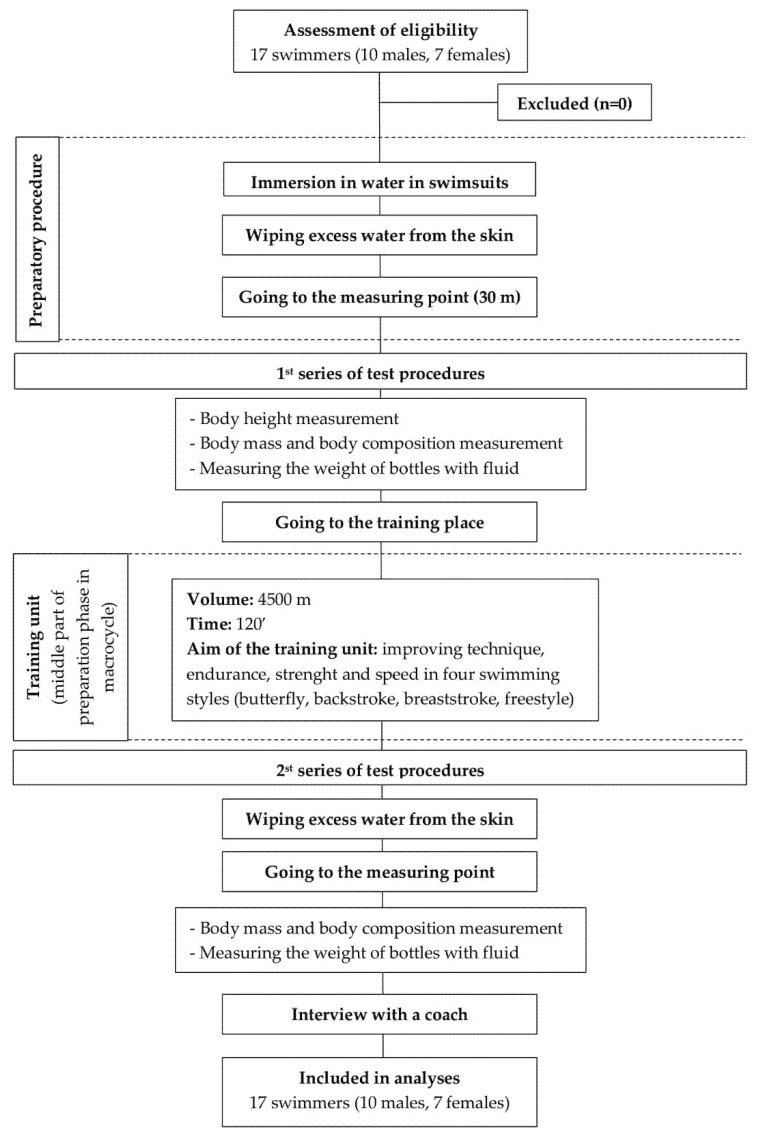
A flow chart of the study design.

The study was conducted with a group of 17 swimmers (seven females (body height: 166 ± 4.3 cm; body mass: 61.2 ± 6.4 kg; body mass index: 22.2 ± 1.7) and 10 males (body height: 179 ± 4.6 cm; body mass: 70.3 ± 7.4 kg; body mass index: 21.9 ± 1.4), Figure 1). Participants were aged 15 to 17 years (16.4 ± 0.6 years) at the time of inclusion. Their training experience was about nine years. The studied swimmers were of a Caucasian race. They were members of a ZSMS Poznan swimming club and were competing in national sport competition in the junior category.

Participation in the study was voluntary; participants were informed about the purpose of the study and could withdraw at any time. An ethical review and approval were waived for this study due to the non-interventional and non-invasive status of this observational preliminary study. The test protocol was conducted in accordance with the ethical standards of the Helsinki Declaration and all participants and their parents gave informed consent to their participation.

### 2.2. Training Characteristics

The athletes performed seven units of classic swimming training and three combined units (i.e., strength training in the gym and then swimming) as standard during the week. This gave an average of about 22.5 h of training and about 50 km of distance covered in the water during the week-long training period. The micro-cycle in which the test was performed took place in the middle of the general preparation period, two months before the first competition held in April that year.

The training unit during which the test took place lasted two hours. During this time, the athletes had to swim a distance of 4500 m, performing exercises to improve technique, endurance, strength and speed in the four most important swimming styles (butterfly, backstroke, breaststroke and freestyle).

### 2.3. Procedures

The study took place in February 2018 at the Olympic swimming pool, during the second swimmer training unit on that day, held between 4 and 6 p.m. The water and air temperature were 27 °C.

After enrolment, the study started with collecting basic information from the young swimmers (name, surname, age). Then, they were asked to immerse themselves in the pool water (so that their swimsuits and hair were soaked in water) and wipe themselves off in order to remove the excess water from their bodies. The next step was to move to a medical room (30 m away), where the height, weight and composition of the body were measured and drink bottles weighed. After the measurements were taken, the competitors went to train. After the training unit, athletes were asked to wipe themselves off again and go to the medical room where the second weight and body composition measurement took place and the drink bottles were weighed again. After the second measurement was taken, information was collected from the swimmers’ coach about the training registration details performed by the athletes. A flow chart of the study design is presented in Figure 1.

### 2.4. Body Mass and Composition Analysis

Body height was measured using an anthropometer (GPM, Zurich, Switzerland). The body weight (BW) and composition (bioimpedance analysis, BIA) of the athletes were measured with the single-frequency (50 kHz, 90 μA) and multi-segmental TANITA BC-418 MA analyser (Tokyo, Japan). This analyser allows for precise measurements within the age range of 7 to 99 years. The same device was used throughout the entire study. After the sex, age and height data were entered into the TANITA BC-418 MA device, swimmers were asked to stand of the weighting platform (upright position) with bare feet. Their toes and heels were contacted with the electrodes of the platform. Afterwards, the participants grasped the device grips with electrodes with both hands and the measurements began. Body composition indices were estimated using the manufacturer’s inbuilt algorithm utilizing regression formulas based on weight, height, age, sex and whole-body resistance. The measurements detected by used device included indicators such as weight, body fat percentage, fat mass, fat free mass, body water mass, and impedance. Furthermore, the test–retest reliability for whole-body lean body mass and body fat percentage estimates were ≥0.945 using the intra-class correlation coefficient.

Drink bottles were weighted using a certified kitchen scale (CLATRONIC KW 3366, accuracy up to 1 g (Kempen, Germany)). In order to avoid any bias resulting from changing the person doing the measurement, the same person evaluated pre- and post-training measurements. During the training unit, swimmers could drink ad libitum the carbohydrate hypotonic drink (3%) UNISPORT, made by Nutrend (Olomouc, Czech Republic). The athletes did not eat solid food. In order to compensate for factors that may affect the difference between the first and second weight measurements other than training, the athletes submerged themselves in the pool water so that their swimsuits and hair were soaked in water. They were also asked not to use the toilet between weight measurements. Due to a possible abnormal bioimpedance analysis result, 24 h before training, athletes were required not to consume alcohol or caffeine. By adjusting swimmers’ BW after training to the amount of fluids they consumed, the actual value of changes in BW that would have occurred after training if swimmers had not consumed fluids during training was also calculated. By subtracting the swimmers’ BW from this value before training, the amount of swimmers’ FL during the training unit was obtained.

### 2.5. Statistical Analysis

The results, presented as means and standard deviations (SD), were analysed using an Excel sheet, which is part of the MS 2015 package (Microsoft, Washington, DC, USA) and Statistica 13.3 software (StatSoft Inc., Tulsa, OK, USA). The normality of data distributions was checked by the Shapiro–Wilk test. A t-test for dependent samples was used to detect statistically significant differences between pre-training and post-training results, while a *t*-test for independent samples was used to detect differences between females’ and males’ FI and FL. Relationships between selected variables were demonstrated by using the Pearson correlation coefficient r. Statistical significance for all analyses was established at the level of 95.0% (*p* < 0.05). The effect size by d Cohen (0.2 to 0.5—small effect, 0.5 to 0.8—medium effect, 0.8 to 1.3—large effect, > 1.3—very large effect) was used to complete the statistical inference for individual comparisons (G*Power 3.1.9.6; Franz Faul, Universität Kiel, Kiel, Germany) [23].

## 3. Results

The results concerning usual FI and actual FL for the whole study group are presented in Table 1. These values were presented to deepen the interpretative possibilities per hour (mL/h), minute (mL/min), 1000 m (mL/1000 m) and per kilogram of BW per hour (mL/kg/h). Significant gender differences between female and male group were revealed in FL (females vs. males: −284 mL vs. −674 mL (*p* = 0.004, d = 1.73), −142 mL/h vs. −337 mL/h (*p* = 0.004, d = 1.69), −63 mL/1000 m vs. −150 mL/1000 m (*p* = 0.004, d = 1.7), −2.2 mL/kg/h vs. −4.8 mL/kg/h (*p* = 0.006, d = 1.61), and −4.7 mL/min vs. −11.2 mL/min (*p* = 0.004, d = 1.69); Table 1). Furthermore, the total average FI and total actual FL were about 531 mL and −513 mL, respectively, for the whole study group. Some noticeable but insignificant gender differences were observed in this respect (FI vs. FL: 469 mL and −284 mL for females; 574 mL and −674 mL for males). In addition, no intergender dependence was observed in FI.

The results concerning BW (before and after exercise with and without fluid intake), TBW, fat free mass (FFM), fat mass (FM) for the whole group, for females and males are presented in Table 2. The results show the values measured in the participants before and after the training unit. Significant differences were observed between BW before and after training without taking fluids for the whole group (66.5 kg vs. 66.0 kg, *p* < 0.001, d = 0.06; Table 2), females (61.2 kg vs. 60.9 kg, *p* = 0.015, d = 0.04; Table 2) and males (70.3 kg vs. 69.6 kg, *p* < 0.001, d = 0.91; Table 2). For TBW and FFM before and after training, significant differences were observed only in males (TBW: 43.8 L vs. 43.2 L, *p* = 0.002, d = 0.14 and 62.4% vs. 61.7%, *p* < 0.001, d = 0.36; FFM: 59.8 kg vs. 59.1 kg, *p* = 0.002, d = 0.12; Table 2).The weight of adipose tissue differed after training in the whole group (12.5 kg vs. 12.9 kg, *p* = 0.032, d = 0.11; Table 2) and in the group of males (10.5 kg vs. 11.1 kg, *p* < 0.001, d = 0.24; 14.8% vs. 15.7%, *p* < 0.001, d = 0.34; Table 2). No significant differences in FM before and after training were observed in the females’ group.

**Figure 2:**
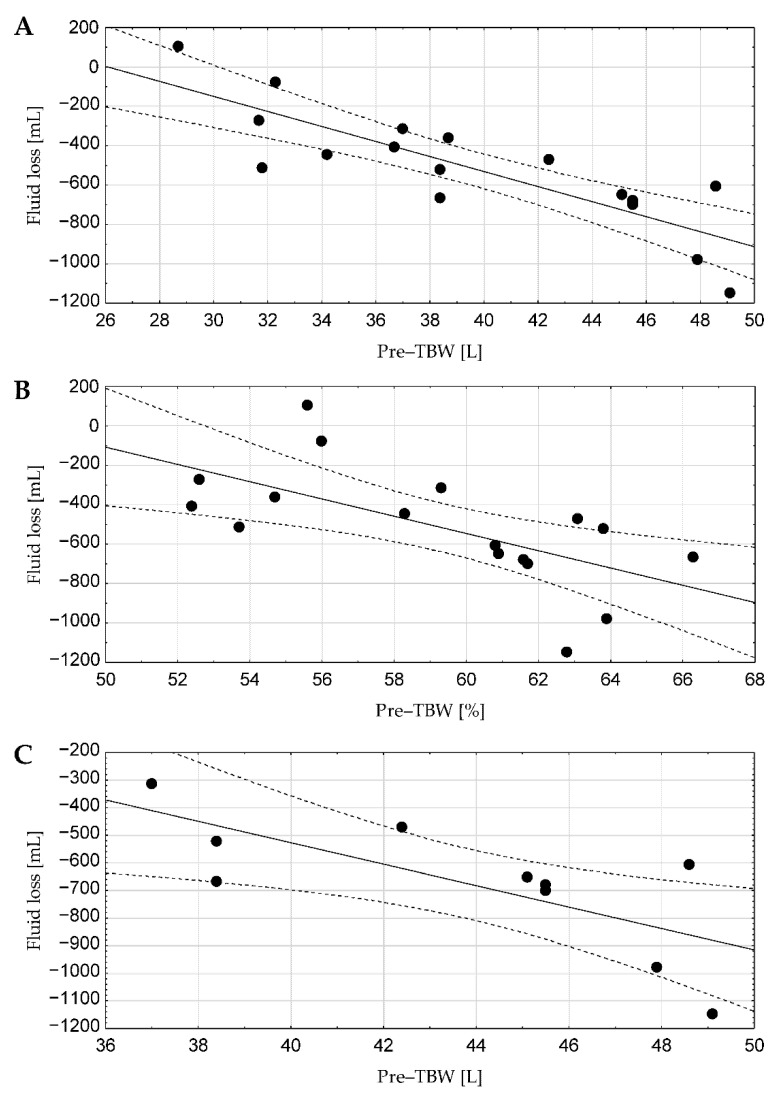
(**A**–**C**) present relationships between actual fluid loss and total body water content before training in the whole study group (mL vs. L (**A**) and mL vs. % (**B**)), also in the group of males (mL vs. L (**C**)). The black dots represent the values of the individual participants.

Evaluation of the relationships between FI and actual FL, the TBW before training and the actual FL and between FI and TBW before training for the whole group, female and male are presented in Table 2 and Figure 2. There were no statistically significant correlations between FI and actual FL and between FI and TBW before training. However, significant correlations were observed in the whole group between actual FL (mL) and pre-workout TBW respectively (in %: r = −0.64; in litres: r = −0.84). In relationship to sex, although no significant correlations were detected in females, males showed a correlation between pre-workout TBW (L) and actual FL (mL) (r = −0.73) (Table 3).

## 4. Discussion

The results of the study carried out in young swimmers provide additional information and fill the scientific gap regarding FL as a result of a standard two-hour swimming training unit, taking into account the usual FI during the training, as well as changes in body composition, and selected correlations of factors that can substantially contribute to athletic performance and competition results. During that training unit, swimmers’ total body water content decreased about 0.5% for females and 1.0% for males.

We registered a large effect size between gender-related FL (d: from 1.61 to 1.72; *p*: from 0.004 to 0.006). This loss in the whole study group was on average −513 mL (about −284 mL for females and −674 mL for males). Taking into account the recorded customary FI, the average body hydration status changes were about −267 mL/h in the whole group (−142 mL/h in females and −337 mL/h in males). These data are lower than those presented by Cox et al. [24] in a study on weight changes and spontaneous FI in water polo athletes (23 males) and swimmers (20 females and 21 males) aged between 16 and 32 years. Moreover, the quoted authors noted that, in swimmers, FL was ~314 mL/h for females and ~415 mL/h for males. However, the lower amount of FL in our studies could result from a lower intensity of muscle work during the training unit. We would like to underline that our investigations took place during the period of general macrocycle preparation, in which the efforts during the given training units are usually characterised by average intensity. Moreover, it should be noted that during training, the swimmers covered a relatively small distance—4500 m. Additionally, consultations with coaches suggested that during a training unit of the same duration as the analysed one (two hours), swimmers can even swim ~33% longer distances. In our opinion, the FL would probably be higher than the one observed and, taking into account mathematical assumptions, if it grew proportionally, it could be about −684 mL for the whole group (about −378 mL for females and about 900 mL for males). On an hourly training basis, these values would be about −342 mL/h for the whole group, about 189 mL/h for females, and about −450 mL/h for males, thus they would be much closer to those observed by Cox and colleagues [24].

In our study, the average FI per training unit was about 531 mL for the entire study group (~469 mL for females and ~574 mL for males). In this range, it corresponded on average to 69% (and 110% and 56.8%, respectively) in relation to the recommendations in this field [25]. Nevertheless, the amount of fluids usually taken by swimmers had an effect on the BW of the athletes after training, which, in the case of the whole group, was comparable to that before training (in the case of females, it was on average 0.2 kg higher; in the case of males, it was 0.1 kg lower). Our results are also similar to those presented in the study of Adams et al. [21] on water balance in teenage swimmers. It can be assumed that changes in BW were insignificant (about 0.1 ± 0.3 kg) due to balanced ad libitum FI.

Therefore, taking into account the recommendations on FI by Domínguez et al. [25] concerning the nutritional needs of swimmers, indicating an optimal water/fluid intake of 150% of the amount of FL during training, the amount of fluids consumed during training in the examined athletes seems to be acceptable for both females and males. Although these values are less than about 150% of the water lost through sweat, this demand does not have to be covered during training only; it can be done after a training unit as well. It is worth noting that this is of fundamental importance, especially if athletes perform two or more training units per day, in order to start their next efforts in a state of euhydration.

Furthermore, the studied swimmers were analysed before and after the second training unit on that day. The optimal pre-training hydration status of this group is indicated by TBW values marked in the body composition analysis. Moreover, during the training of the tested swimmers, the average BW of the whole group did not change significantly after exercise (in the case of females it was even slightly higher, and in the case of males it was slightly lower). This is beneficial in practice because maintaining the state of optimal hydration allows for the effort to be carried out with maximum physiological capacity through advantageous effects on endurance performance [26] and cognitive performances [27], as well as avoiding the risk of the negative effects of dehydration that can be observed in athletes already with very low FL of one to two percent of BW (which may already be manifested by an increased heart rate, internal temperature or muscle glycogen usage) [28]. However, it should also be mentioned that in some cases FI may not exert a spectacular impact on some of the exercise results achieved (especially those related to physical abilities, such as power or speed) [29]. In a study of 19 swimmers aged 11 to 17, Briars et al. [29] recorded that the supply of mineral water, isotonic drinks or lack of FI, respectively, did not improve specific swimming performance (results of 50 m sprints), although during a training unit in water (lasting over 105 min) among swimmers who did not receive any fluids, a reduction in TBW of at least a 0.42% was observed.

In addition, the results obtained in our study indicate that while maintaining the current practice of hydration, no negative changes resulting from excessive FL and/or dehydration should be observed in the examined young swimmers. However, it is worth recalling that the performed training unit was neither high in volume nor in intensity. In this respect, it seems reasonable to assume that in case of swimmers who had swum longer distance and/or exercised with higher intensity, the reduction of hydration could have been more elevated than reported in our study. Furthermore, as indicated in another study [21], water has a much higher thermal capacity, thermal conductivity and density than air, making the heat exchange much faster. It seems that these factors may affect the rate of thermoregulation in water, which may additionally vary with water temperature [30]. Therefore, given that pool water temperatures range from 25 to 28 °C [2], it is possible that a different water temperature than that observed in our study (27 °C) could cause a different rate of FL.

Simultaneously, it is worth briefly noting that the FL during training performed in a land environment among athletes of other disciplines was also analysed. The results of Broad et al. [31] show that the average FL during training in summer was about 1371 mL/h for basketball players and 985 mL/h for football players. Although training in a water environment, according to the opinions of some coaches and athletes, apparently seems to have less impact on the disturbance of optimal hydration of swimmers than the training of players of other disciplines performed in a land environment, it is still fundamental to properly and regularly hydrate swimmers before, during and after training, as emphasized by the authors of the current recommendations [25]. This thesis also seems to be confirmed by the important relationship observed in this study between the TBW before training and the actual FL in young swimmers (in the whole group and the group of males), where higher TBW correlated with higher FL during exercise. However, no correlation between the TBW before training and actual FL was observed in females. This may be due to a slightly smaller number of females in the study group and may also hypothetically suggest that they were training in a state of suboptimal hydration, as this state results in a lower FL through sweat for thermoregulation than in a state of optimal hydration [32]. Moreover, our investigation was carried out during the second training unit of the day, which could have affected the swimmers’ hydration state before the second training. A valuable indicator that would help to assess the hydration status before the training would be the evaluation of osmolality and urinary-specific gravity [33]. To confirm the reasonableness of these proceedings, Adams et al. [21], using these indicators, have recorded that 67% of swimmers started their first training unit in an inadequate state of hydration. For these reasons, we are convinced that regular body composition monitoring during both the preparatory and competitive season should be recommended as a useful tool to support athletic training.

Although it may be puzzling to note the recorded increase in the fat mass of the swimmers tested (on average before and after training, respectively: ~10.5 kg vs. ~11.1 kg in the case of males, and ~12.5 kg vs. ~12.9 kg in the whole group), according to the authors, the reason for such results lies in the accuracy of the applied measurement methods (BIA). As a result of medium- and high-intensity physical exercise, the value of the measured impedance changes, which in turn leads to a disruption in repeatability and a decrease in the accuracy of the test method. Consequently, results show a significant measurement error (values before vs. after exercise). Moreover, the change in hydration itself significantly affects the FFM and FM indications based on the bioimpedance method. This problem has been described in more detail in a study by Dehghan and Merchant [34] on the accuracy of electrical bioimpedance in human population studies and has also been extensively raised in studies using bioimpedance analysis to determine body composition in athletes [35,36]. Nevertheless, using bioimpedance analysis for measuring body water changes and fat-free mass can be a useful method that confirms Utter and Lambeth’s study regarding evaluation of the accuracy of multifrequency bioelectrical impedance analysis on body composition in high school wrestlers [37] and seems to be easier and more practical for the coach and athletes than other methods such as evaluation of osmolality and urinary-specific gravity. However, with this method, strict assurance of the recommended measurement conditions is essential.

Finally, we would also like to point out some limitations of our study, which should be taken into account in the interpretation of the presented results. Although well-selected methods of measurement and analysis have been used, some limitations should be mentioned here, such as the small size of the study group (especially female (*n* = 7)) and the body composition analysis methods (restrictions related to, e.g., the risk of measurement artifacts due to a gravitational effect on body fluids in a standing position, potential difficulties with the one-way comparison of the results with other BIA analysers (different BIA technologies and frequencies may produce different outputs), dependence of the final body composition value on the equation formulas used, and possibility of impedance influenced by exercise or beverage intake [38,39,40]). Furthermore, the possibility of interpretation being limited to the specific protocol of the training unit (the influence of exercise work intensity and environmental conditions, and, as we described above, that our studies were carried out before and after the second training unit on that day), the lack of studies on specific hydration markers (such as osmolality and urinary-specific gravity) [33], and the lack of possibility to measure the amount of water that could be absorbed by the skin. Interestingly, the latter aspect may be clinically relevant, as it was noted many years ago that by simply immersing the body in water, up to about 400 mL of water can be absorbed through the skin in one hour [41]. Furthermore, more recent studies involving swimmers have similarly indicated that this phenomenon may affect the final results [29], which is indirectly confirmed by the observations in our study in one of the swimmers, whose BW increased by 300 g after training, despite her consumption of only 198 mL of fluids.

The authors would like to underline that the final results may also be affected by the difficulty of making a meaningful assessment of the amount of water that may be accidentally swallowed in the course of the field study of swimming. Breaststroke, butterfly and freestyle swimming, where the exhalation takes place under water and the inhalation must take place very quickly (when the swimmer’s mouth is above the water surface for a very short time), may lead to the involuntary swallowing of water [42]. It is worth noting that a lower BW after training can also result to a small extent from depletion of glycogen reserves. Its content in the human body is about 600 g, most of which is located in the muscles [43], and as a result of intensive and long-term physical effort, the content of glycogen in active muscle cells may decrease by up to about 10% of the initial values [44]. Nonetheless, at the same time, it should be pointed out that undeniable advantages of our study are a homogeneous group of analysed subjects, as well as the fact that field study measurements were conducted during customary training in identical conditions using the same certified equipment, and with precise monitoring of the fluids actually consumed, the distance travelled, and changes in BW, FL and TBW.

## 5. Conclusions

The present study indicates a relationship between fluid loss and pre-training total body water content and that swimming training in an aquatic environment is associated with fluid loss that seems slightly lower than in a land environment. However, there is also a risk of dehydration in swimmers, especially when they train at a higher volume and intensity, or at a higher temperature; therefore, it is important to pay particular attention to effective hydration procedures during training units, taking into account all external factors, to prevent its negative effects on health and performance.

## Figures and Tables

**Table 1:** Fluid intake and actual fluids loss in the whole group, female and male.

Variable		All (*n* = 17)	Females (*n* = 7)			Males (*n* = 10)			*p-*Value *^*	Cohen’s d Effect Size
		Mean ± SD	Mean	±	SD	Mean	±	SD	Females vs. Males	Females vs. Males
Fluid intake	mL	531 ± 322	469	±	310	574	±	340	0.525	0.3227
	mL/h	266 ± 161	235	±	155	287	±	170	0.525	0.3197
	mL/1000 m	118 ± 72	104	±	69	128	±	76	0.525	0.3306
	mL/kg/h	4.1 ± 2.4	3.9	±	2.5	4.2	±	2.5	0.810	0.1200
	mL/min	8.9 ± 5.4	7.8	±	5.2	9.6	±	5.7	0.525	0.3299
Actual fluids loss	mL	−513 ± 300	−284	±	221	−674	±	240	0.004 *	1.7259
	mL/h	−257 ± 150	−142	±	111	−337	±	120	0.004 *	1.6870
	mL/1000 m	−114 ± 67	−63	±	49	−150	±	53	0.004 *	1.7046
	mL/kg/h	−3.7 ± 2.0	−2.2	±	1.8	−4.8	±	1.4	0.006 *	1.6125
	mL/min	−8.6 ± 5.0	−4.7	±	3.7	−11.2	±	4.0	0.004 *	1.6870

Values are expressed as means ± SD. ^ difference between two independent means—females vs. males. Effect size by d Cohen (0.2 to 0.5—small effect, 0.5 to 0.8—medium effect, 0.8 to 1.3—large effect, >1.3—very large effect); * statistically significant.

**Table 2:** Body mass and composition before (pre) and after (post) workout in the whole group, female and male.

Variable			All (*n* = 17)			Females (*n* = 7)					Males (*n* = 10)				
			Mean ± SD	*p-*Value *^*	Cohen’s d Effect Size *^*	Mean	±	SD	*p-*Value *^*	Cohen’s d Effect Size *^*	Mean	±	SD	*p-*Value *^*	Cohen’s d Effect Size *^*
Body mass	kg	Pre	66.5 ± 8.5			61.2	±	6.9			70.3	±	7.8		
		Post_TOT_	66.5 ± 8.3	0.861	0.0000	61.4	±	6.8	0.149	0.0292	70.2	±	7.6	0.497	0.0130
		Post_WFI_	66.0 ± 8.3	<0.001 *	0.0595	60.9	±	6.8	0.015 *	0.0438	69.6	±	7.6	<0.001 *	0.9089
TBW	L	Pre	39.5 ± 6.6	0.139	0.0314	33.4	±	3.4	0.076	0.0895	43.8	±	4.5	0.002 *	0.1377
		Post	39.3 ± 6.1			33.7	±	3.3			43.2	±	4.2		
	%	Pre	59.3 ± 4.4	0.052	0.0719	54.8	±	2.1	0.180	0.0975	62.4	±	2.0	<0.001 *	0.3586
		Post	59.0 ± 3.9			55.0	±	2.0			61.7	±	1.9		
FFM	kg	Pre	54.0 ± 9.0	0.144	0.0344	45.7	±	4.6	0.058	0.0652	59.8	±	6.1	0.002 *	0.1175
		Post	53.7 ± 8.4			46.0	±	4.6			59.1	±	5.8		
FM	kg	Pre	12.5 ± 3.7	0.032 *	0.1124	15.5	±	3.0	0.168	0.0339	10.5	±	2.4	<0.001 *	0.2447
		Post	12.9 ± 3.4			15.4	±	2.9			11.1	±	2.5		
	%	Pre	19.1 ± 6.0	0.065	0.0704	25.3	±	2.9	0.083	0.1403	14.8	±	2.7	<0.001 *	0.3394
		Post	19.5 ± 5.3			24.9	±	2.8			15.7	±	2.6		

Values are expressed as means ± SD. Means: difference between two dependent means; ^ in reference to “pre” value, effect size by d Cohen (0.2 to 0.5—small effect, 0.5 to 0.8—medium effect, 0.8 to 1.3—large effect, >1.3—very large effect); FFM–Fat Free Mass, FM–Fat Mass, Post_TOT_–post exercise including fluid intake, Post_WFI_–post exercise without fluid intake, TBW–Total Body Water content; * statistically significant.

**Table 3:** Correlation between selected primary indices in the whole group, female and male.

Variable			R	R^2^	t	*p-*Value
Fluid Intake vs. Actual Fluids Loss	mL vs. L	All (*n* = 17)	−0.14	0.02	−0.55	0.590
		Females (*n* = 7)	−0.41	0.17	−1.02	0.356
		Males (*n* = 10)	0.17	0.03	0.48	0.646
Pre-exercise Total Body Water content vs. Actual Fluids Loss	% vs. mL	All (*n* = 17)	−0.64	0.41	−3.20	0.006 *
		Females (*n* = 7)	0.19	0.04	0.43	0.683
		Males (*n* = 10)	−0.35	0.12	−1.05	0.323
	L vs. mL	All (*n* = 17)	−0.84	0.70	−5.90	<0.001 *
		Females (*n* = 7)	−0.59	0.35	−1.65	0.161
		Males (*n* = 10)	−0.73	0.53	−3.01	0.017 *
Fluids Intake vs. Pre-exercise Total Body Water content	mL vs. %	All (*n* = 17)	0.17	0.03	0.68	0.506
		Females (*n* = 7)	0.37	0.14	0.89	0.416
		Males (*n* = 10)	−0.14	0.02	−0.40	0.699
	mL vs. L	All (*n* = 17)	0.05	0.00	0.20	0.846
		Females (*n* = 7)	0.07	0.00	0.16	0.882
		Males (*n* = 10)	−0.23	0.05	−0.67	0.519

Values are expressed as correlation coefficients (R), coefficients of determination (R^2^) and test statistics (t); * statistically significant.

## Data Availability

The datasets used and/or analysed during the current study are available from the corresponding author on reasonable request.
